# The Involvement of Histone H3 Acetylation in Bovine Herpesvirus 1 Replication in MDBK Cells

**DOI:** 10.3390/v10100525

**Published:** 2018-09-27

**Authors:** Liqian Zhu, Xinyi Jiang, Xiaotian Fu, Yanhua Qi, Guoqiang Zhu

**Affiliations:** 1 College of Veterinary Medicine, Yangzhou University, Yangzhou 225009, China; jxy0492@163.com (X.J.); fxt950828@163.com (X.F.); 2 Jiangsu Co-Innovation Center for Prevention and Control of Important Animal Infectious Diseases and Zoonoses, Yangzhou 225009, China; 3 College of Life Science, Zhengzhou University, Zhengzhou 450066, China; yanhuaqi2007@163.com

**Keywords:** BoHV-1, HAT, HDAC, proteasome, histone H3

## Abstract

During bovine herpesvirus 1 (BoHV-1) productive infection in cell cultures, partial of intranuclear viral DNA is present in nucleosomes, and viral protein VP22 associates with histones and decreases histone H4 acetylation, indicating the involvement of histone H4 acetylation in virus replication. In this study, we demonstrated that BoHV-1 infection at the late stage (at 24 h after infection) dramatically decreased histone H3 acetylation [at residues K9 (H3K9ac) and K18 (H3K18ac)], which was supported by the pronounced depletion of histone acetyltransferases (HATs) including CBP/P300 (CREB binding protein and p300), GCN5L2 (general control of amino acid synthesis yeast homolog like 2) and PCAF (P300/CBP-associated factor). The depletion of GCN5L2 promoted by virus infection was partially mediated by ubiquitin-proteasome pathway. Interestingly, the viral replication was enhanced by HAT (histone acetyltransferase) activator CTPB [*N*-(4-Chloro-3-trifluoromethylphenyl)-2-ethoxy-6-pentadecylbenzamide], and vice versa, inhibited by HAT inhibitor Anacardic acid (AA), suggesting that BoHV-1 may take advantage of histone acetylation for efficient replication. Taken together, we proposed that the HAT-dependent histone H3 acetylation plays an important role in BoHV-1 replication in MDBK (Madin-Darby bovine kidney) cells.

## 1. Introduction

Bovine herpesvirus 1 (BoHV-1) is an important pathogen that causes pneumonia, conjunctivitis, genital disorders, and abortions in cattle [1]. BoHV-1 infection induces severe inflammatory response though diverse mechanisms, such as by overexpression of pro-inflammatory cytokines and reactive oxidative species [2,3,4]. The suppression of host immune response by virus infection may render secondary infection by diverse pathogens, such as bovine viral diarrhea viruses (BVDV), bovine respiratory syncytial virus (BRSV), parainfluenza-3 virus (PI3V), bovine coronaviruses, *Mannheimia haemolytica*, *Pasteurella multocida*, *Histophilus somni* and *Mycoplasma* spp. [5,6], and consequently lead to a life-threatening pneumonia known as bovine respiratory disease complex (BRDC), one of the costliest ailments in cattle feeding [7,8].

In eukaryotes, DNA is packaged into a protein-DNA complex called chromatin, with nucleosome as monomeric subunit containing a core of histone proteins (H2A, H2B, H3, and H4) surrounding by ~147 bp of genomic DNA [9]. The chromatin is dynamically organized into regions of either loosely packaged actively transcribed chromatin (euchromatin) or highly condensed transcriptionally repressed chromatin (heterochromatin) through diverse epigenetic modifications, such as by acetylation, methylation, ubiquitination, phosphorylation, and sumoylation [10,11,12,13]. The acetylation of certain lysine (K) residues in histones H3 and H4 is generally an indicator of transcriptionally active chromatin [14,15].

Increasing evidence has elucidated the implication of epigenetic modification either in viral gene transcription or in viral productive infection. For example, during HSV-1 productive infection histone H3 associates with viral DNA at the IE(immediate early) promoters, thereby recruiting the chromatin remodeling factors into viral replication compartments [16,17,18,19], which facilitates viral gene expression and DNA replication. The acetylation of histones on parvoviral DNA is essential for viral gene expression and completion of the viral life cycle [20]. Histone acetylation is essential for influenza A virus infection, since the inhibition of histone acetylation by histone acetyltransferase (HAT) inhibitors can attenuate its infection [21]. Histone is also involved in BoHV-1 infection because BoHV-1 infection decreases histone H4 acetylation [22], and a portion of intranuclear viral DNA is present in nucleosomes [23], and histone H4 is found to be packaged into virions [24]. However, the role of histone H3 acetylation in BoHV-1 productive infection is still not fully defined.

In this study, the status of histone H3 acetylation, the potential mechanisms for the modification, as well as its role in BoHV-1 infection in MDBK cells were investigated. For the first time we demonstrated that virus infection significantly reduced histone H3 acetylation, which correlated well with the pronounced depletion of HATs including CBP/P300 (CREB binding protein and p300), GCN5L2 (general control of amino acid synthesis yeast homolog like 2) and PCAF (P300/CBP-associated factor). Moreover, histone acetylation contributed to viral gene expression. Therefore, we concluded that HAT-dependent histone H3 acetylation plays an important role in BoHV-1 replication in MDBK cells.

## 2. Materials and Methods

### 2.1. Cells and Virus

MDBK (Madin-Darby bovine kidney) cells (kindly provided by Dr. Leonard J. Bello, University of Pennsylvania) were maintained in DMEM (Thermo Fisher Scientific, Waltham, MA, USA) supplemented with 10% horse serum (HyClone Laboratories, Logan, UT, USA). BoHV-1 of Colorado1 stain (kindly provided by Dr. Leonard J. Bello, University of Pennsylvania) was propagated in MDBK cells. Aliquots of virus stocks were stored at −70 °C until use. The inactivation of the BoHV-1 virus with UV (ultraviolet) irradiation was performed as previously described [25]. Complete inactivation of the virus was characterized by plaque assay in MDBK cells.

### 2.2. Antibodies and Reagents

CBP/p300 rabbit mAb (monoclonal antibody) (Cat#7389, 1:1000), PCAF rabbit mAb (Cat#3378,1:1000), GCN5L2 rabbit mAb (Cat#3305, 1:1000), Histone H3 rabbit mAb (Cat#4499, 1:1000), Acetyl-Histone H3 (Lys9) rabbit mAb (Cat#9649, 1:1000), Acetyl-Histone H3 (Lys18) rabbit mAb (Cat#13998, 1:1000), ubiquitin Mouse mAb(Cat#3936, 1:1000), HDAC1 (histone deacetylas) mouse mAb (Cat#5356, 1:1000), HDAC2 mouse mAb (Cat#5113, 1:1000), HDAC3 mouse mAb (Cat #3949, 1:1000), HDAC4 rabbit mAb (Cat #7628, 1:1000), β-actin rabbit mAb(Cat#4970, 1:1000), HRP (horseradish peroxidase) labeled anti-mouse IgG (Cat#7076, 1:3000) and HRP labeled anti-rabbit IgG (Cat#7074, 1:3000), were purchased from Cell Signaling Technology (Beverly, MA, USA). BoHV-1 VP16 antibody (1:2000) is kindly provided by Prof. Vikram Misra at the University of Saskatchewan [26].

Anacardic acid (AA) (Cat#A7236), trichostatin A (TSA) (#8552). MG132 (Cat#474791-1), ammonium chloride (NH4Cl) (Cat#254134), were ordered from Sigma-Aldrich (St. Louis, MO, USA). Bortezomib (#S1013) was obtained from selleckchem.com (Houston, TX, USA). *N*-(4-Chloro-3-trifluoromethyl-phenyl)-2-ethoxy-6-pentadecyl-benzamide (CTPB) (Cat#586976-24-1) was provided by Santa Cruz Biotechnology (Dallas, TX, USA).

### 2.3. Cytotoxicity Assays by Trypan-Blue Exclusion Test

Cytotoxicity of indicated chemicals in MDBK cells was assessed by Trypan-blue exclusion test, as described by Fiorito et al. [27,28], with modification. In brief, MDBK cells in 24-well plates were treated with or without chemicals at indicated concentrations for 24 h. Then the cells were collected by trypsinization, and an aliquot of the cell suspension was mixed with an equal volume of 0.4% Trypan-blue (0.4%) (Bio-Rad, Hercules, CA, USA, #1450021). After incubation for 10 min, cells were counted using a Burker chamber under a light microscope. The percentage of cell viability in the chemical treatment groups was calculated by normalization of the number of live cells to that in the control samples. The value of cell viability in the control was arbitrarily set to 100%.

### 2.4. Western Blotting Analysis

Confluent MDBK cells in 60 mm dishes were infected with BoHV-1(MOI = 1) for 4, 12 and 24 h. Cell lysates were prepared using lysis buffer (1% Triton X-100, 50 mM sodium chloride, 1 mM EDTA, 1 mM EGTA, 20 mM sodium fluoride, 20 mM sodium pyrophosphate, 1 mM phenylmethylsulfonyl fluoride, 0.5 g/mL leupeptin, 1 mM benzamidine, and 1 mM sodium orthovanadate in 20 mM Tris–HCl, pH 8.0). To test the effects of certain inhibitors on the designated signaling, MDBK cells were infected for 24 h along with treatment with indicated chemicals at the designated concentrations. Cell lysates were prepared using lysis buffer as described above.

Cell lysates were separated on 8 or 10% SDS–polyacrylamide gels, and proteins were transferred to a polyvinylidene difluoride (PVDF) membrane (Bio-Rad, Hercules, CA, USA). Targeted proteins were detected using respective antibodies. The intensity of immune reactive bands was analyzed with free software image J (https://imagej.nih.gov/ij/download.html). To calculate the relative protein expression levels, the band intensity of target proteins was firstly normalized to β-actin, then normalized to the control lane.

### 2.5. Immunoprecipitation (IP) Assay

For IP studies, MDBK cells in 60-mm dishes were infected with BoHV-1 at an MOI of 1. At 16 h after infection, cells were lysed with 600 mL of RIPA buffer (1× PBS, 1% NP-40, 0.5% sodium deoxycholate, 0.1% SDS) supplemented with protease inhibitor as described above in Western blots analysis. Cell lysates were clarified by centrifugation at 12,000 rpm for 20 min, and incubated with Dynabeads^®^ (Life Technologies, Carlsbad, CA, USA, Cat. No. 10001D), which have been incubated with 4 µL of Acetyl-Histone H3 (Lys9) rabbit mAb (Cell Signaling Technology, Cat#9649) or GCN5L2 rabbit mAb (Cell Signaling Technology, Cat#3305) for 1 h at room temperature with rotation. After overnight incubation at 4 °C with rotation, the beads were collected with the help of a magnet (DynaMag™) (Life Technologies, Cat. No. 12321D). After three washing with PBS, beads were boiled in SDS loading buffer and Western blots were performed to detect the designated proteins.

### 2.6. Virus Replication Inhibition Assay

MDBK cell in 24-well plates were infected with BoHV-1 (MOI of 1) along with the treatment of indicated chemicals(PAA, Anacardic acid, TSA, and CTPB) at the designated concentration for 1 h at 37 °C, After three washing with PBS, fresh medium with designated chemicals was added to each well. At 24 h after infection, viral yields were titrated in MDBK cells. The cell cultures treated with DMSO was used as a control. The results are expressed as TCID_50_/mL calculated using the Reed-Muench formula.

### 2.7. Quantification of mRNA by qRT-PCR

Confluent MDBK cells in 60 mm dishes were infected with BoHV-1 using an MOI of 1. At 4, 8 and 16 h post infection(hpi) total RNA was purified with TRIzol LS Reagent (Ambion, Thermo Fisher Scientific, Waltham, MA, USA, Cat#10296010) following the manufacturers’ instructions. Freshly prepared RNA (1 μg) was used as a template for the synthesis of the first-strand cDNA with commercial random hexamer primers for viral mRNA detection using Thermoscript™ RT-PCR system Kit (Invitrogen, Carlsbad, CA, USA, Cat#11146-024). The cDNA products were used as templates for relative qRT-PCR to measure levels of viral mRNA of bICP4, and bICP22 as well as cellular gene glyceraldehyde-3-phosphate dehydrogenase (GAPDH) with specific primers as previously described in the reference [29]. Analysis of GAPDH mRNA was used as an internal control. Relative qRT-PCR was carried out using the ABI 7500 fast real-time system (Applied Biosystems, Foster City, CA, USA). Separate GAPDH amplification was used to normalize gene expression. The data were analyzed using the equation 2^−ΔΔ*C*T^ method.

## 3. Results

### 3.1. BoHV-1 Infection of MDBK Cells Decreases Histone H3 Acetylation

**Figure 1:**
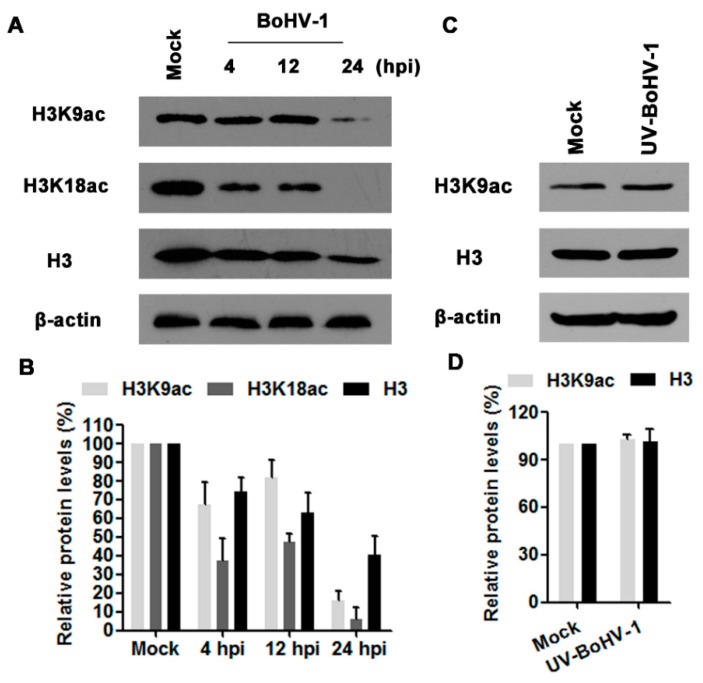
The effects of BoHV-1 infection on histone H3 acetylation. (**A**) MDBK (Madin-Darby bovine kidney) cells in 60 mm dishes were mock infected or infected with BoHV-1 at a MOI of 1 for 4, 12 and 24 h. The cell lysates were then prepared for Western blots to detect histone H3, H3K9ac and H3K18ac. Data shown are representative of three independent experiments. (**C**) MDBK cells in 60 mm dishes were mock infected or infected with UV (ultraviolet)-inactivated BoHV-1 at an MOI of 1 for 24 h. The cell lysates were prepared and subjected to Western blots to detect histone H3 and H3K9ac. Data shown are representative of three independent experiments. (**B**,**D**) The band intensity was analyzed with software image J. Each analysis was compared with that of uninfected control which was arbitrarily set as 100%. The error bars denote the variability between the three independent experiments.

Acetylation of histone H3 is involved in transcription activation, and H3K9ac is an epigenetic marker for histone acetylation. To test whether BoHV-1 infection alters histone H3 acetylation, we evaluated the expression levels of H3K9ac, H3K18ac as well as H3 in virus infected MDBK cells at 4, 12, and 24 hpi. As a result, virus infection consistently decreased the expression levels of H3K9ac, H3K18ac and H3, and peaked at 24 hpi (Figure 1A). Quantitative analysis indicated that the levels of H3K9ac, H3K18ac and H3 were decreased to 15.8%, 6.0% and 40.7% relative to the control, respectively(Figure 1B), suggesting that BoHV-1 infection reduced histone H3 acetylation (H3K9ac and H3K18ac).

Ultraviolet (UV) light-inactivated viruses are replication deficient because it could bind to the receptors and enter the cells, but are unable to express viral genes [25,30]. To further understand whether complete viral replication cycle was required to affect the histone H3 acetylation, UV-inactivated viral particles were employed for further investigation. As shown in Figure 1C,D, UV-inactivated virus had no effects on histone H3 acetylation. Thus, these results suggested that *de novo* viral protein production and/or DNA replication seems to be associated with the decreased acetylation of histone H3.

### 3.2. BoHV-1 Infection Differentially Affects the Expression of HATs and HDACs

**Figure 2:**
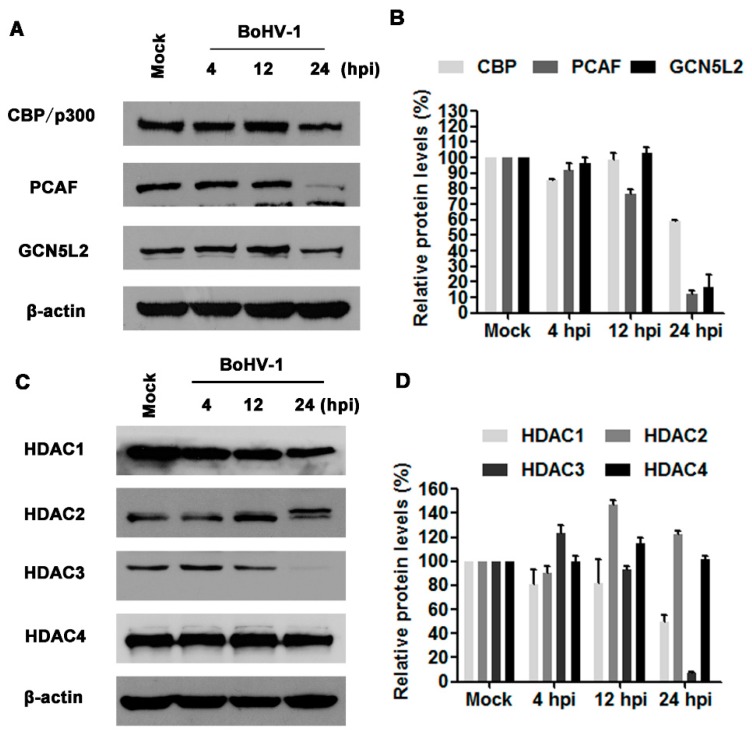
The effects of BoHV-1 infection on the expression of HATs (histone acetyltransferase) and HDACs (histone deacetylases). (**A**) MDBK cells in 60 mm dishes were mock infected or infected with BoHV-1 at an MOI of 1 for 4, 12 and 24 h. The cell lysates were then prepared for Western blots to detect (CREB binding protein and p300), GCN5L2 (general control of amino acid synthesis yeast homolog like 2) and PCAF (P300/CBP-associated factor). Data shown are representative of three independent experiments. (**B**,**D**) The relative band intensity was analyzed with software image J, and each analysis was compared with that of uninfected control which was arbitrarily set as 100%. The error bars denote the variability between the three independent experiments. (**C**) MDBK cells in 60 mm dishes were infected with BoHV-1 at an MOI of 1 for 4, 12 and 24 h. The cell lysates were then prepared for Western blots to detect HDAC1, HDAC2, HDAC3 and HDAC4. Data shown are representative of three independent experiments.

Histone acetylation and deacetylation are reversible processes regulated enzymatically by HATs and histone deacetylases (HDACs). HATs such as CBP/p300, GCN5L2, and PCAF, are enzymes that acetylate conserved lysine residues on histones. To understand the mechanisms underlying the decreased histone H3 acetylation by virus infection, we initially detected the protein levels of CBP/p300, PCAF, and GCN5L2 following BoHV-1 infection at 4, 12, and 24 hpi. Virus infection altered the expression of CBP/p300, PCAF, and GCN5L2, only at 24 hpi, all of them were robustly decreased in comparison to the mock infected control (Figure 2A). The protein levels of CBP/p300, PCAF, and GCN5L2 were reduced to approximately 59.3%, 12.5%, and 16.4% relative to the control, respectively (Figure 2B). The global decease of HATs expression at 24 h after infection may reflect their reduced capability for histone acetylation, which is in agreement with the decreased histone H3 acetylation.

HDACs are a family of enzymes controlling deacetylation of histones. The family of mammalian HDACs is comprised of at least 18 members which are classified into four classes: class I (HDAC1, 2, 3 and 8), class II (HDAC 4, 5, 6, 7, 9 and 10), class III (SIRT 1 to 7) and class IV (HDAC11) [31]. In this study, the expression of HDAC1-4 in response to virus infection was detected using Western blots. As can be seen in Figure 2C,D, virus infection altered the expression of both HDAC1 and HDAC3 with distinct manners, while neither HDAC2 nor HDAC4 were apparently affected. At 24 hpi, the expression levels of HDAC1 and HDAC3 were decreased to approximately 49.6% and 6.8% relative to the control, respectively. The unexpected decreased expression levels of both HDAC1 and HDAC3 may undermine the finding that virus infection decreased the acetylation of histone H3. Taken together, virus infection altered the expression of HATs and HDACs with distinct manners. Relative to HDACs, the prominently decreased expression of HATs strongly supported the reduced acetylation of histone H3 at 24 hpi.

### 3.3. The HAT Inhibitor Limits BoHV-1 Replication

**Figure 3:**
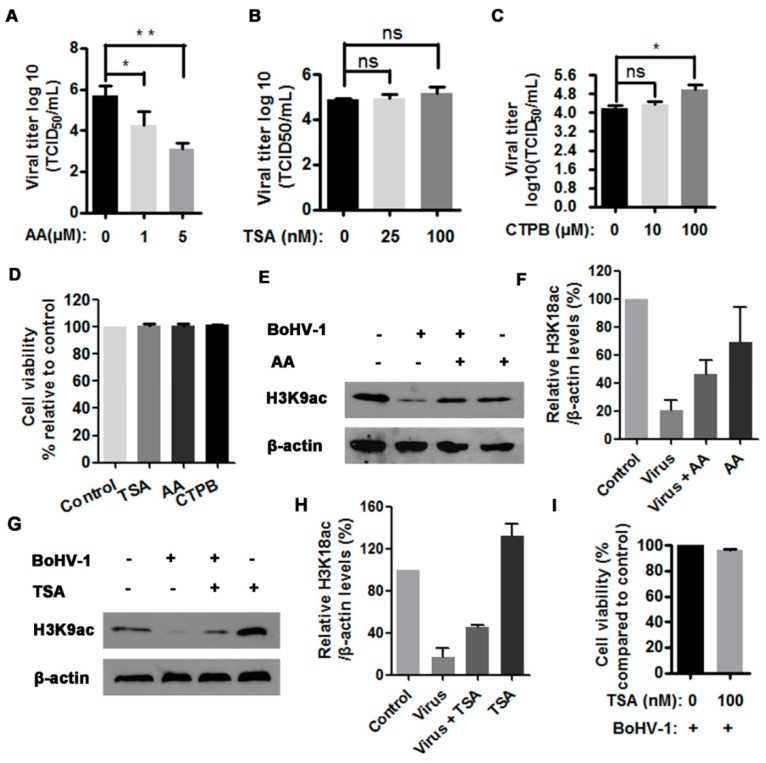
The effects of HAT inhibitor on BoHV-1 productive infection. (**A**–**C**) MDBK cells in 24-wells plates were infected with BoHV-1 (MOI = 1) and treated with anacardic acid (AA) (0, 1 and 5 μM) (**A**), TSA (trichostatin A) (0, 50 and 100 μM) (**B**), CTPB [*N*-(4-Chloro-3-trifluoromethylphenyl)-2-ethoxy-6-pentadecylbenzamide] (0, 10 and 100 μM) (**C**) or DMSO control for 1 h, respectively. After three washing with PBS, fresh medium with either inhibitors or DMSO control were replaced. At 24 hpi, viral yields were determined in MDBK cells. Data represent three independent experiments. Significance was assessed with the student *t* test (* *p* < 0.05, ** *p* < 0.01, ns: not significant). (**D**) The cytotoxicity of AA (5 μM), TSA (100 μM), and CTPB (100 μM) was analyzed in MDBK cells with Trypan-blue exclusion test. Data represent means of three independent experiments. (**E**,**G**) MDBK cells in 60 mm dishes were uninfected or infected by BoHV-1 at an MOI of 1, along with the treatment of either AA (5 μM) (**E**) or TSA (100 nM) (**G**), or DMSO control. At 16 hpi, cell lysates were prepared and subjected to Western blots to detect the expression of H3K9ac. Data represent three independent experiments (+: indicated compound or virus was present, −: indicated compound or virus was not present). (**I**) The virus infected MDBK cells were mock treated with DMSO or TSA (100 nM) throughout infection. At 24 hpi, the cell viability was detected with Trypan-blue exclusion test. Data represent means of three independent experiments. (**F**,**H**) The band intensity was analyzed with software image J. Each analysis was compared with that of uninfected control which was arbitrarily set as 100%. The error bars denote the variability between the three independent experiments.

Our foregoing results demonstrated that virus infection differentially altered HATs and HDACs expression, particularly the depletion of HATs correlated with the reduced histone H3 acetylation. Therefore, the role of HATs and HDACs in BoHV-1 productive infection was independently investigated using HAT inhibitor anacardic acid (AA) and HDAC inhibitor Trichostatin A (TSA), respectively. AA specifically inhibits the enzymatic activity of HATs, such as CBP/p300 and PCAF, and thereby affects HAT-dependent gene transcription [32]. Also, AA has been reported to have multiple other biological effects such as antitumor activity and antioxidant activity [33]. In this study, we found that the treatment of virus-infected cells with HAT inhibitor AA at a concentration of 1 μM and 5 μM resulted in a 1.4- and 2.6-log reduction of the virus titer comparing to that in the mock-treated control, respectively (Figure 3A). Indeed, 5 μM of AA treatment could inhibit histone H3 acetylation as demonstrated by the reduced levels of H3K9ac relative to the control, but AA increased the levels of H3K9ac in the context of virus infection in comparison to the mock treated but infected cells (Figure 3E,F). Maybe the significantly decreased progeny virus by AA treatment led to rescuing the depletion of H3K9ac attributed to virus infection.

TSA is an HDAC-specific inhibitor that can selectively inhibit the enzymatic activities of class I and II HDACs, but not class III HDACs [34]. Interestingly, it was reported that influenza virus infection decreased HDAC1 expression in A549 cells, and the treatment of infected cells with 1 and 5 μM of TSA resulted in 3.1-fold and 5.3-fold increase of progeny virus relative to the control, respectively [35]. In this study, we used much fewer concentrations of TSA to investigate its role in BoHV-1 replication. We found that the treatment with 100 nM of TSA could restore histone H3 acetylation (Figure 3G,H), but had no impact on the viral replication because the virus titer was only increased ~0.2 log (equal to 2-fold), with a difference not statistically significant (*p* > 0.05) (Figure 3B). Of note, all the concentrations used for indicated inhibitors had no cytotoxicity to MDBK cells (Figure 3E). But we noticed that in the context of virus infection the cell viability was reduced to approximately 96.2% by TSA (100 nM) treatment (Figure 3I), which is a possible reason for why TSA treatment could not evidently booster viral replication. These results suggested that the maintenance of HAT activities was essential for virus productive infection.

CTPB is a potent activator of CBP/p300, but not of PCAF activities. To further confirm the role of HAT played in the virus infection, CTPB, was employed for further investigation. CTPB at a concentration of 100 μM showing no cytotoxicity to MDBK cells can significantly promote virus productive infection (Figure 3C,E). The virus titer was increased ~0.9 log by the treatment with 100 μM of CTPB in comparison with the mock-treated control (Figure 3C). This observation further suggested that the histone acetylation by HAT played an essential role in BoHV-1 productive infection.

### 3.4. The HAT Inhibitor Affects Viral Gene Expression

**Figure 4:**
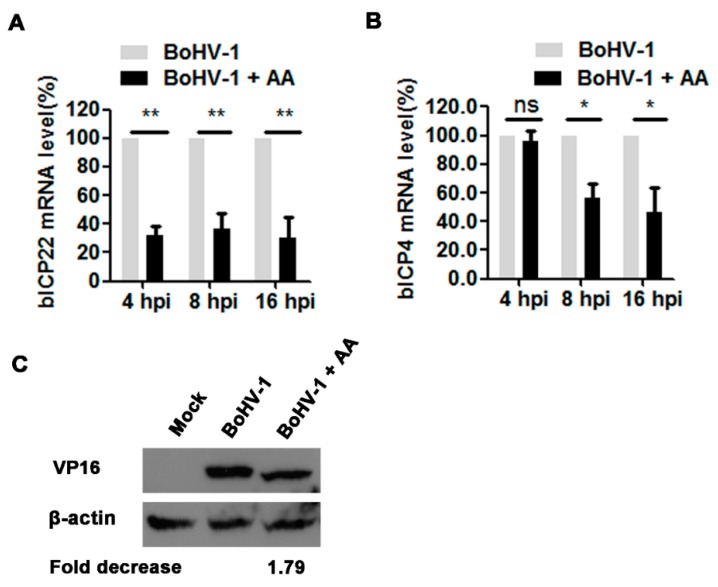
The effects of HAT inhibitor on BoHV-1 gene expression. (**A**,**B**) The virus infected cells were treated with DMSO or AA (5 μM). Total RNA was prepared at indicated time points, and qRT-PCR was performed to determine the mRNA levels of bICP4 (**A**) and bICP22 (**B**). (**C**) MDBK cells in 60 mm dishes were infected by BoHV-1 at an MOI of 1 with the treatment of DMSO or AA (5 μM), at 16 hpi, cell lysate was prepared and subjected to Western blots to detect VP16 protein. The band intensity was analyzed with software image J. And analysis was compared with that of untreated but infected control. Data represent three independent experiments. Significance was assessed with the student *t* test (* *p* < 0.05, ** *p* < 0.01). ns: not significant.

Considering that histone acetylation regulated by HAT is an important factor controlling gene expression, we further investigated the effects of chemical inhibition of HATs on viral gene expression. For this purpose, the virus-infected cells were treated with 5 μM of AA or DMSO as a control, and the mRNA levels of immediate early (IE) genes including bICP4 and bICP22 were detected with relative qRT-PCR. In AA treated virus-infected cell cultures, the mRNA levels of bICP4 decreased to 56.0% and 46.7% relative to the mock-treated control, at 8 and 16 hpi, respectively, but at 4 hpi the chemical treatment showed minor effects (Figure 4A). BICP22 mRNA levels were reduced to 32.1%, 37.1% and 30.0% by AA treatment relative to the control samples, at 4, 8 and 16 hpi, respectively (Figure 4B). Though these IE proteins were not detected due to the unavailability of given antibodies, these results of qRT-PCR indicated that the HAT inhibitor AA affected the transcription of these IE genes.

An additional study was performed to examine the effects of AA on the expression of viral tegument protein VP16 by Western blots using an antibody against VP16. As demonstrated in Figure 4C, at 16 hpi VP16 protein expression levels decreased approximately 1.79-fold by the treatment with AA relative to that in the mock-treated control, indicating that AA affected VP16 expression. In summary, these findings further suggested that this HAT inhibitor affected BoHV-1 productive infection in MDBK cells, and therefore the maintaining of HAT activity is essential for virus efficient replication.

### 3.5. The Proteasome Pathway—Mediated GCN5L2 Degradation Is Potentially Involved in BoHV-1 Infection-Decreased Histone H3 Acetylation

**Figure 5:**
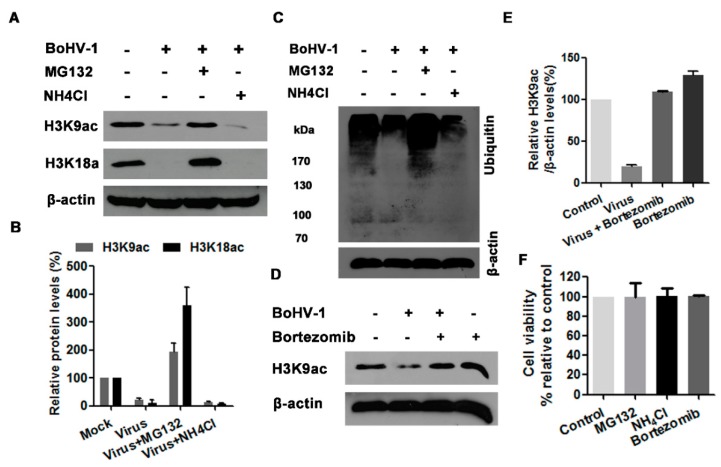
The effects of ubiquitin-proteasome pathway on histone H3 acetylation. (**A**,**C**) MDBK cells in 60 mm dishes were mock infected or infected with BoHV-1 (MOI = 1) and treated with either MG132 (1 μM) or NH4Cl (10 mM), or mock treated with DMSO vehicle for 24 h. The cell lysates were prepared for Western blots to detect the expression of H3K9ac and H3K18ac (**A**), and the ubiquitined protein (**C**). Data shown are representative of three independent experiments. (**D**) MDBK cells in 60 mm dishes were infected with BoHV-1 (MOI = 1) and treated with bortezomib (2 nM), or mock treated with DMSO control for 24 h. The cell lysates were prepared for Western blots to detect the expression of H3K9ac. Data shown are representative of three independent experiments. (**B**,**E**) The band intensity was analyzed with software image J. Each analysis was compared with that of uninfected control which was arbitrarily set as 100%. The error bars denote the variability between the three independent experiments. (**F**) The cytotoxicity of MG132 (1 μM), ammonium chloride (NH4Cl) (10 mM) and bortezomib (2 nM) in MDBK cells for 24 h was analyzed by Trypan-blue exclusion test. Data represent the means of three independent experiments. +: indicated compound or virus was present, −: indicated compound or virus was not present.

Generally, there are two potential pathways to control protein degradation in eukaryotic cells, one mediated by ubiquitin-proteasome and other mediated by lysosome [36], which can be efficiently inhibited by chemical inhibitors, such as MG132 and NH4Cl, respectively. To identify whether the ubiquitin-proteasome and/or lysosome pathways are involved in the reduced acetylation of histone H3 at 24 h after infection, the virus-infected MDBK cells were treated with either proteasome inhibitor MG132 or lysosome inhibitor NH_4_Cl throughout infection as determined elsewhere [35,37]. The treatment of MG132 (1 μM) reversed the depletion of both H3K9ac and H3K18ac attributed to virus infection, and rescued their expression to a level higher than that in the uninfected control, whereas NH_4_Cl treatment did not show any effect (Figure 5A,B). The increased levels of ubiquitinated proteins in MG132-treated cells but not in NH_4_Cl-treated cells confirmed the efficiency of MG132 as an inhibitor for the proteasome pathway in MDBK cells (Figure 5C). Though the concentration of MG132 used in this study did not show obvious cytotoxicity to MDBK cells (Figure 5F), the chemical may have off-target effects. So, another proteasome inhibitor bortezomib was employed to validate the rescued effects of MG132. As expected, bortezomib at a concentration of 2 nM showing no cytotoxicity to MDBK cells could reverse BoHV-1-reduced expression of histone acetylation marker H3K9ac to a level a little bit higher than that in the uninfected control (Figure 5D–F), though bortezomib showed relative lower capability than MG132. These results suggested that the ubiquitin-proteasome pathway may contribute to the decreased acetylation of histone H3 in the virus infected cells.

**Figure 6:**
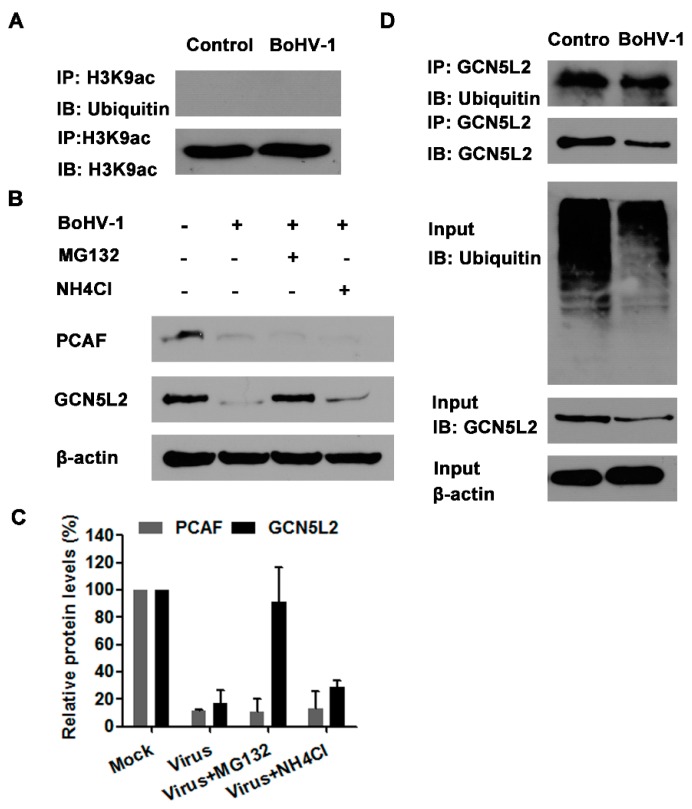
The ubiquitin-proteasome pathway mediated GCN5L2 degradation. (**A**,**D**) MDBK cells in 60 mm dishes were mock infected or infected with BoHV-1 (MOI = 1) for 24 h. The cell lysates were prepared for IP using the antibody of either against H3K9ac (**A**) or GCN5L2 (**D**). The IP samples were subjected to immunoblots using antibodies against ubiquitin, H3K9ac and GCN5L2. The expression of GCN5L2 and ubiquitinated proteins in the input cell lysates in panel D were detected as a control**.** Data shown are representative of three independent experiments. (**B**) MDBK cells in 60 mm dishes were infected with BoHV-1 (MOI = 1) and treated with MG132 (1 μM), or mock treated with DMSO control for 24 h. The cell lysates were prepared for Western blotting to detect the expression of PCAF and GCN5L2. Data shown are representative of three independent experiments. (**C**) The band intensity was analyzed with software image J. Each analysis was compared with that of uninfected control which was arbitrarily set as 100%. The error bars denote the variability between the three independent experiments. +: indicated compound or virus was present, −: indicated compound or virus was not present.

In view that the proteasome inhibitors of both MG132 and bortezomib could rescue the depletion of H3K9ac (a marker for histone H3 acetylation) attributed to virus infection, we investigated whether H3K9ac was ubiquitinated in the cells with or without infection by IP assay. Unexpectedly, when we performed IP with the H3K9ac specific monoclonal antibody, the ubiquitinated protein bands could not be detected in the cells with or without infection using ubiquitin specific antibody (Figure 6A), indicating that H3K9ac is not ubiquitinated in MDBK cells. So it was highly possible that the depletion of acetylated histone H3 due to virus infection was not caused by the proteasome-mediated H3K9ac degradation. 

Our foregoing results indicated that both PCAF and GCN5L2 were significantly decreased by the virus infection (Figure 2A). Interestingly, the treatment of virus-infected cells with 1 μM of MG132 could significantly reverse the depletion of GCN5L2 but not PCAF (Figure 6B,C). We speculated that virus infection may promote GCN5L2 degradation via proteasome pathway. Therefore, IP was performed with GCN5L2 specific monoclonal antibody, and ubiquitin specific antibody was used to detect the ubiquitination of GCN5L2 in the cell cultures. As a result, the ubiquitinated GCN5L2 could be detected from the IP sample by the immunoblot using ubiquitin specific antibody (Figure 6D). This result indicated that virus infection might target GCN5L2 for ubiquitin-mediated degradation, which would partially account for the depletion of acetylated histone H3 and correlated with the reversed depletion of H3K9ac by the treatment of MG132 in the virus-infected cells.

## 4. Discussion

Acetylation is one of the best-characterized covalent modifications of histones. Hyperacetylation of histones is associated with an “open chromatin” conformation and transcriptional activation, whilst hypoacetylation of histones is associated with condensed chromatin and gene silencing [38,39]. For some DNA viruses, such as HSV-1 and canine parvovirus, much is known about the effects of histone modification on virus replication. During HSV-1 productive infection, the viral genomes are associated with histones immediately after injection into the nucleus, and viral proteins ICP0 and VP16 are required to enhance histone acetylation on the viral genome to enable efficient viral gene expression [18,40]. During canine parvovirus infection, cellular histones are associated with viral DNA, and histone acetylation on parvoviral DNA is essential for viral gene expression [20]. BoHV-1 tegument protein VP22 associates with histones and thereby decreased histone H4 acetylation in infected cells or VP22 transfected cells [22]. In this study, for the first time we demonstrated that histone H3 acetylation (H3K9ac and H3K18ac) was significantly decreased during BoHV-1 infection in MDBK cells, and peaked at 24 h after infection (Figure 1), which suggested the involvement of histone H3 acetylation in BoHV-1 infection.

To further elucidate the potential mechanisms for the decreased histone H3 acetylation in response to the virus infection, the steady state expression of certain HATs and HDACs were investigated because they regulate enzymatically histone acetylation with opposite effects. We found that the virus infection led to a global decrease of all the detected HATs, including CBP/p300, GCN5L2 and PCAF (Figure 2A), which was clearly in favor of the finding that virus infection decreased histone H3 acetylation (Figure 1). However, it seems that downregulation of both HDAC1 and HDAC3 by virus infection was not correlated with the decreased levels of histone H3 acetylation, which emphasized the complexity of the mechanisms for the regulation of histone acetylation following virus infection. It has been reported that HDAC1 regulates influenza A virus (IAV) replication independent of its deacetylation activity [41]. HDAC1 stimulates host type I interferon antiviral response, therefore IAV infection decreases HDAC1 expression for efficient replication [35]. The treatment with TSA, an HDAC inhibitor, increases IAV infection via the inhibition of signal transducer and activator of transcription I (STAT1) pathway, and the depression of interferon-stimulated genes including IFITM3, ISG15, and viperin in IAV-infected cells [35]. HDAC3 is required for inflammatory gene expression in response to LPS stimulation [42]. So, HDAC1 and HDAC3 can stimulate host antiviral response or inflammatory response, which tend to be depressed by virus infection. Moreover, HDAC inhibitors promote HSV-1 productive infection in neural cells [43,44]. Likewise, we found that TSA enhanced BoHV-1 virus yield approximately 2-fold, but the difference was not statistically significant (Figure 3). Therefore, we assumed that the downregulation of HDAC1 and HADC3 would be beneficial for BoHV-1 efficient replication independent of their deacetylation activity, which needs further extensive studies, in the future.

It has been reported that the ubiquitin-proteasome pathway, generally known to mediate protein degradation, is involved in BoHV-1 productive infection and immune invasion [28,45]. Here, we found that the ubiquitin-proteasome inhibitors could reverse the depletion of acetylated H3 in BoHV-1 infected cells (Figure 3). Mechanistically, the virus infection targeted GCN5L2 but not acetylated histone H3 for proteasome-mediated degradation (Figure 6), which may consequently reduce the acetylation of histone H3. Previous studies have reported that some BoHV-1-encoded viral proteins promote the proteasome-mediated degradation of certain cellular proteins. For example, both interferon response factor 3 (IRF3) and promyelocytic leukemia (PML) are targeted by viral proteins bICP0 for proteasome-dependent degradation [45,46]. Viral UL49.5 protein is involved in the proteasome-mediated degradation of the transporter associated with antigen presentation [47]. The BoHV-1 host shutoff protein UL41 destabilizes the expression of immune responses related genes by ubiquitin-proteasome pathway [48]. Taken together, the above evidence indicates that virus-encoded proteins may target specific cellular proteins for proteasome-dependent degradation. Whether a specific viral protein(s) is involved in the depletion of GCN5L2 through proteasome pathway is an interesting subject which is remained to be determined in the future.

In this study, we found that BoHV-1 replication was positively correlated with HAT activity because the treatment with HAT activator (CTBP) increased the virus yield, and vice versa, it was significantly decreased by HAT inhibitor (AA) partially through affecting virus gene expression(Figure 3 and Figure 4). This finding is supported by a previous report that CBP/p300, an HAT, enhances BoHV-1 productive infection and transactivation of late viral protein gC promoter [49]. Taken together, these findings suggested that BoHV-1 infection may take advantage of histone acetylation for efficient replication. We speculated that HAT-dependent histone H3 acetylation plays an important role in BoHV-1 replication in MDBK cells.

In this study, the expression status of acetylated H3 including H3K9ac and H3K18ac was investigated in the context of virus infection. We demonstrated that histone acetylation played an important role in BoHV-1 replication, while the virus infection decreased histone H3 acetylation by differentially altered expression of HATs and HDACs. In addition, virus infection targeted GCN5L2 for degradation via the proteasome pathway, which correlated well with the reversed depletion of H3K9ac by the treatment of MG132 in the virus-infected cells. We suggested that the HAT-dependent histone (H3) acetylation plays an important role in BoHV-1 replication in MDBK Cells

## 5. Conclusions

In summary, we provided evidence that BoHV-1 infection decreased histone H3 acetylation in MDBK cells, while histone acetylation played an important role in BoHV-1 replication. We suggested that HAT-dependent acetylation of histone H3 plays a vital role in BoHV-1 replication. This finding might add our knowledge on understanding the mechanism for the viral pathogenesis.
